# Development of Simultaneous Analytical Method of Three Polypeptide Toxins α‐Amanitin, β‐Amanitin and Phalloidin in Poisonous Mushrooms and Human Serum Using UHPLC–MS/MS

**DOI:** 10.1002/jms.5145

**Published:** 2025-05-21

**Authors:** Hang‐Ji Ok, Eun‐Young Park, Yongho Shin, Jeong‐Han Kim, Min‐Ho Song, Ji‐Ho Lee

**Affiliations:** ^1^ Department of Agricultural Biotechnology, College of Agriculture and Life Sciences Seoul National University Seoul Korea; ^2^ Department of Technical Research Center Shimadzu Scientific Korea Seoul Korea; ^3^ Department of Food Science and Technology University of California, Davis Davis California USA; ^4^ Department of Applied Bioscience Dong‐A University Busan Korea; ^5^ School of Natural Resources and Environment Science, College of Agriculture and Life Sciences Kangwon National University Chuncheon Korea

**Keywords:** mushroom, quantitative analysis, serum, toxins, UHPLC–MS/MS

## Abstract

Accidental ingestion of toxic mushrooms remains a global public health concern because of the presence of highly potent peptide toxins such as α‐amanitin, β‐amanitin, and phalloidin. These compounds exhibit strong hepatotoxicity and can lead to acute liver failure and death. However, their rapid detection in biological and food matrices remains analytically challenging. Existing methods often require extensive sample preparation and are not suitable for urgent diagnostic applications. This study presents the development and validation of a rapid and sensitive analytical method for the simultaneous quantitation of α‐amanitin, β‐amanitin, and phalloidin in poisonous mushrooms and human serum. Among several preparation strategies evaluated, a method following direct extraction with 1% formic acid in methanol was selected for its speed, simplicity, and effectiveness in minimizing matrix interference. The method demonstrated excellent linearity (*r* ^2^ ≥ 0.99), low quantitation limits (10–50 ng/mL), and satisfactory recovery (72%–117%) and precision (RSD ≤ 19%) in both food and biological matrices. When applied to field‐collected *Amanita virosa*, α‐amanitin and β‐amanitin were detected at 39 and 145 mg/kg, respectively, whereas no toxins were found in *Amanita volvata*. These findings demonstrate that the established method enables rapid and reliable detection of lethal peptide toxins with minimal sample preparation. The protocol is suitable for forensic investigations, clinical toxicology, and food safety monitoring. Its applicability in emergency settings underscores its potential as a practical tool for improving public health responses to mushroom poisoning incidents.

## 1 Introduction

For centuries, mushrooms have been consumed as a low‐calorie dietary component rich in plant‐derived proteins, essential minerals (e.g., selenium, potassium, and copper), and vitamins (e.g., B complex and vitamin D) [1]. In several regions, mushrooms have also been used in traditional medicine [1, 2]. Approximately 5000 mushroom species are estimated to exist globally, although only 20%–25% have been formally described, and over 100 species are classified as poisonous [3, 4, 5]. Toxic mushrooms often resemble edible varieties in morphology, which frequently leads to accidental ingestion by untrained foragers, resulting in severe poisoning or death.

Numerous cases of mushroom poisoning have been reported worldwide. Between 2001 and 2010, Japan recorded 569 incidents involving 1920 patients and 10 deaths [6]. In Northern Italy, 160 cases associated with the consumption of *Amanita phalloides* occurred during 1981, resulting in 4 fatalities [7]. In Korea, 5 poisoning cases were reported between 2014 and 2023, with 38 individuals affected. Overall, the global mortality due to toxic mushroom ingestion is estimated at 200–250 deaths annually [1].

**FIGURE 1:**
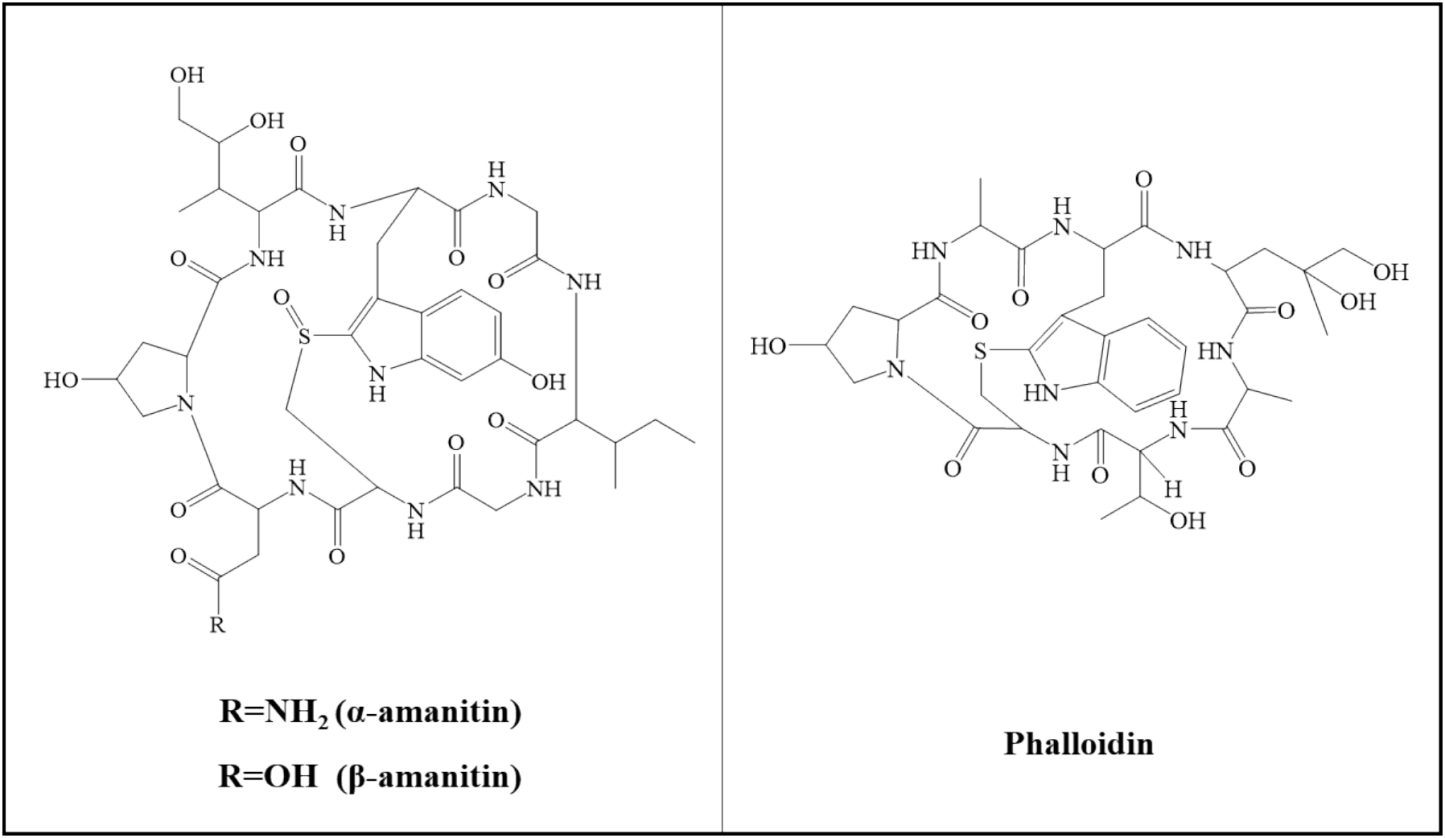
Structures of amanitins and phalloidin.

Among the toxic species, *Amanita virosa* and *Amanita volvata* are particularly lethal because of their production of amatoxins (α‐amanitin and β‐amanitin) and phallotoxins (phalloidin) [8, 9]. These toxins have distinct chemical structures, with amatoxins forming bicyclic octapeptides and phallotoxins forming bicyclic heptapeptides (Figure 1). Amatoxins act as selective inhibitors of RNA polymerase II, halting protein synthesis and inducing apoptosis [3]. These compounds exert severe toxicity, targeting hepatic, renal, and central nervous tissues [10, 11, 12]. The oral LD_50_ of α‐amanitin and β‐amanitin in rats ranges from 0.2 to 0.6 mg/kg, and the estimated human LD_50_ for α‐amanitin is 0.1 mg/kg [13, 14]. Although phallotoxins are less toxic, they still demonstrate strong hepatotoxicity, with an intraperitoneal LD_50_ of 2.0 mg/kg in mice [15, 16, 17]. These toxins remain detectable in serum for approximately 30 h and in urine for up to 72 h after ingestion [8, 18].

Accurate identification of these toxins is critical for effective medical treatment; however, symptom‐based diagnosis is often unreliable in emergency situations. Rapid and quantitative detection in both mushroom tissues and biological matrices is essential for applications in clinical, forensic, and environmental toxicology. Although various studies have focused on the analysis of mushroom toxins [19, 20, 21, 22, 23, 24, 25], many existing methods require lengthy sample preparation involving solid‐phase extraction (SPE) and evaporation, limiting their utility in time‐sensitive scenarios.

Biological matrices such as saliva, plasma, urine, sweat, and hair have been explored for toxin detection [19, 20, 21, 26, 27, 28], with serum preferred because of its homogeneity and lower matrix complexity [29, 30, 31]. Traditional spore identification methods have been largely replaced by instrumental techniques such as high‐performance liquid chromatography (HPLC) [22, 25, 32] and liquid chromatography coupled with mass spectrometry (LC–MS or LC–MS/MS) [22, 25, 32, 33, 34, 35, 36]. Although peptide toxin quantitation from *Amanita* species has been reported in several countries, no such data are currently available for mushrooms collected in Korea [17, 37, 38].

This study aimed to develop and validate a rapid and sensitive UHPLC–MS/MS method for quantifying α‐amanitin, β‐amanitin, and phalloidin in *Amanita virosa*, *Amanita volvata*, and human serum. The method utilizes small sample volumes (200 mg of mushroom and 100 μL of serum) and enables direct detection without extensive cleanup. The proposed approach offers a practical analytical platform for food toxicology, clinical diagnostics, and forensic investigations.

## 2 Materials and Methods

### 2.1 Chemicals and Materials

Three certified reference standards (purity ≥ 98.0%) of α‐amanitin, β‐amanitin, and phalloidin were obtained from Sigma‐Aldrich (St. Louis, MO, USA). HPLC‐grade methanol was purchased from Merck (Darmstadt, Germany), whereas LC–MS grade methanol and formic acid were acquired from Merck (Darmstadt, Germany) and Sigma‐Aldrich (St. Louis, MO, USA), respectively. Dispersive SPE (dSPE) kits containing C18 (25 mg C18 and 150 mg MgSO_4_) and C18/PSA (25 mg C18, 25 mg PSA, and 150 mg MgSO_4_) were purchased from Sigma‐Aldrich (St. Louis, MO, USA). Oasis PRiME HLB cartridges (3 cc, 60 mg) were obtained from Waters Corporation (Milford, MA, USA). Fresh *Lentinula edodes* (shiitake) mushrooms were purchased from a local market and used as a blank matrix. Specimens of *Amanita virosa* and *Amanita volvata* were collected from a natural habitat in Korea. Human serum (male AB plasma, of US origin, and sterile filtered) was supplied by Sigma‐Aldrich (St. Louis, MO, USA). All mushroom samples and serum were stored at −80°C prior to sample preparation.

### 2.2 Preparation of Solvent and Matrix‐Matched Standards

Stock solutions (100 000 ng/mL) of α‐amanitin, β‐amanitin, and phalloidin were individually prepared in methanol, with concentrations adjusted according to compound purity. Working standard solutions in the range of 5–500 ng/mL were obtained by serial dilution of the combined stock solution with methanol.

For matrix‐matched calibration, 20 μL of working standard solution was mixed with 80 μL of control mushroom extract prepared by the final sample preparation procedure, yielding calibration levels from 1 to 100 ng/mL. All stock and working solutions were stored at −20°C until analysis.

### 2.3 UHPLC–MS/MS Analytical Conditions

Quantitative analysis was performed using a Shimadzu LCMS‐8060 triple quadrupole mass spectrometer (Kyoto, Japan) coupled with a UHPLC system comprising a degassing unit (DGU‐20A5R), solvent delivery module (LC‐30ad), autosampler (SIL‐30AC), and column oven (CTO‐20A). Separation was achieved on a Kinetex C18 column (100 × 2.1 mm, 2.6 μm, Phenomenex, Torrance, CA, USA) maintained at 40°C.

The mobile phases consisted of (A) 5‐mM ammonium formate with 0.1% formic acid in water and (B) 5‐mM ammonium formate with 0.1% formic acid in methanol. The gradient elution program for Mobile Phase B was as follows: 5% (0 min), 55% (0.5–2.0 min), 95% (2.0–8.0 min), 100% (8.0–11.0 min), and return to 5% (12.0–16.0 min). The flow rate was set at 0.2 mL/min, and the total run time was 16 min.

A heated electrospray ionization (ESI) source operating in fast polarity switching mode (positive/negative) was employed for ionization. Argon was used as the collision gas for tandem MS fragmentation. Instrument control and data processing were performed using LabSolutions software (Version 5.72, Shimadzu).

For optimization of multiple reaction monitoring (MRM) parameters, standard solutions (2000 μg/mL) were directly infused (4 μL) without a column. Full scan spectra (m/z 100–1000) were acquired, and the most intense precursor ion for each compound was selected and fragmented under various collision energies to obtain two product ions per analyte.

### 2.4 Comparison of Sample Preparation Methods

#### 2.4.1 General Setup for Method Comparison

Shiitake mushroom samples were pulverized under dry ice using a mechanical grinder (FM‐909T, Hanil Science, Seoul, Korea). A standard mixture containing three target toxins was then spiked into the homogenized mushroom powder at a concentration of 50 ng/g.

**FIGURE 2:**
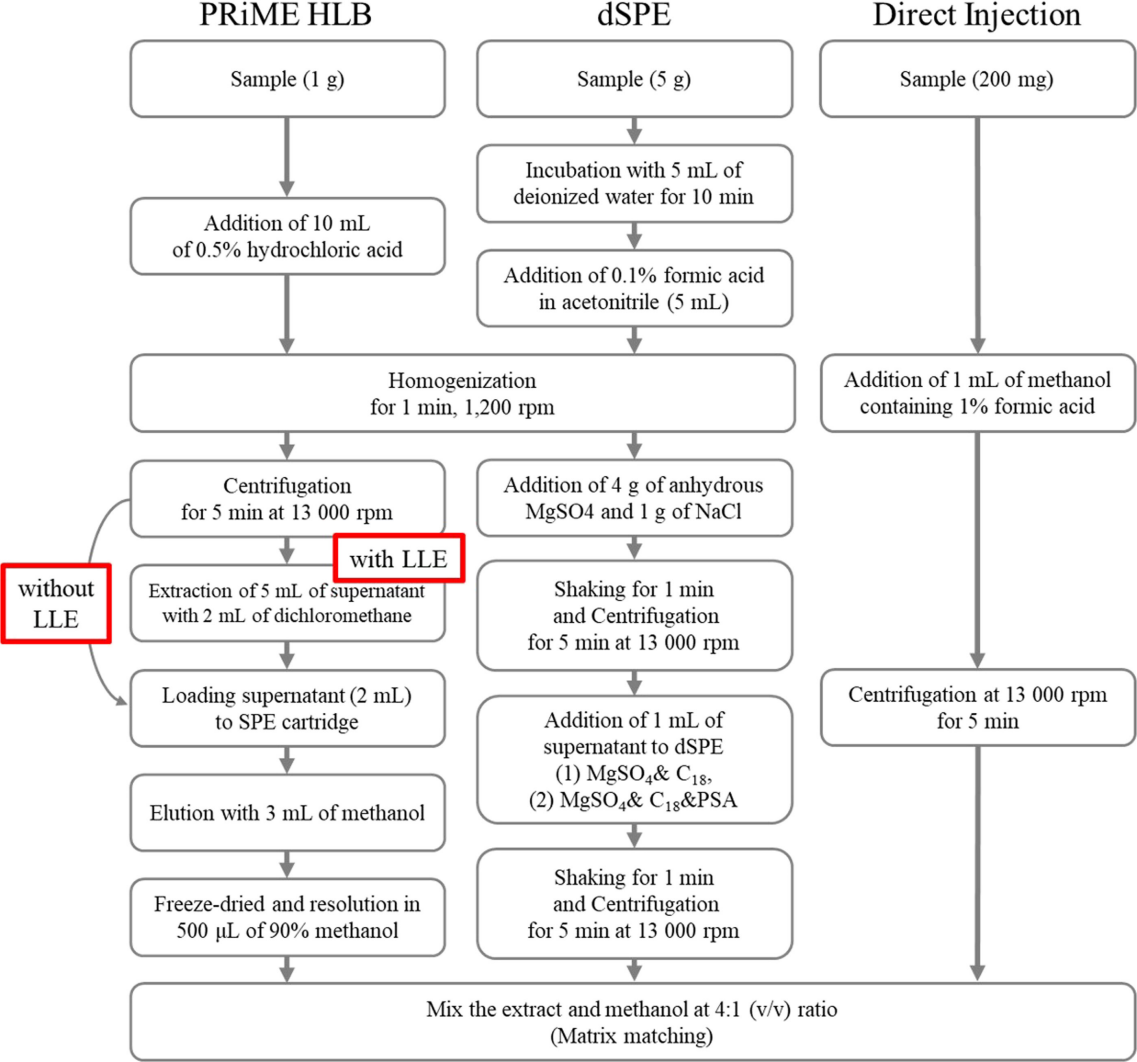
Schematic comparison of the five sample preparation methods evaluated in this study: (1) dSPE with C18 sorbent, (2) dSPE with C18/PSA mixture, (3) PRiME HLB cartridge with and (3) without liquid–liquid extraction (LLE), and (4) direct injection.

Five sample preparation techniques were evaluated: (1) dSPE using C18 sorbent, (2) dSPE using C18/PSA sorbent mixture, (3) PRiME HLB with and (4) without liquid–liquid extraction (LLE), and (5) direct injection without purification. Each procedure was performed according to Figure 2. Different initial sample amounts were selected based on the characteristics of each sample preparation method and the moisture content of the mushroom samples. Every method was repeated three times, and detailed procedures were described below.

#### 2.4.2 PRiME HLB Cartridge With and Without LLE

One gram of homogenized blank sample was used instead of the 0.2‐g dried sample cited in a previous study, after moisture content (~80%) adjustment [39]. The sample was spiked with the mixed toxin standard, and 10 mL of 0.5% hydrochloric acid in water was added. The mixture was shaken at 1200 rpm for 1 min using a tissue homogenizer (1600 MiniG, SPEX SamplePrep, Metuchen, NJ, USA) and centrifuged at 3500 rpm for 5 min.

For the LLE‐based procedure, 5 mL of supernatant was collected and extracted with 2 mL of dichloromethane. The upper aqueous phase (2 mL) was applied to an Oasis PRiME HLB cartridge (3 cc, 60 mg).

For the procedure without LLE, 2 mL of aqueous supernatant, obtained directly after centrifugation, was directly loaded onto the cartridge.

In both procedures, the analytes were eluted with 3 mL of methanol. The eluate was freeze‐dried to near dryness and then reconstituted in 500 μL of methanol–water (10:90, v/v). The final extract was mixed with a blank mushroom matrix extract at a 4:1 ratio prior to LC–MS/MS injection.

#### 2.4.3 dSPE C18 and dSPE C18/PSA Methods

Five grams of fresh sample was used with 5 mL of extraction solvent based on the standard 1:1 extraction protocol for mushrooms [40, 41]. Five milliliters of deionized water was added for 10 min. Acetonitrile containing 0.1% formic acid (5 mL) was added for extraction and shaken for 1 min on a tissue homogenizer at 1200 rpm. To minimize the heat caused by MgSO_4_ and moisture, the centrifuged tubes were cooled down under the ice bath, and then 4 g of anhydrous MgSO_4_ and 1 g of NaCl were added into the tube before shaking for 1 min. After being centrifuged at 3500 rpm (5 min), the supernatant (1 mL) was treated with general dSPE C18 (25 mg C18 and 150 mg MgSO_4_), which did not contain PSA, and dSPE C18/PSA (25 mg C18, 25 mg PSA, and 150 mg MgSO_4_) for comparison with the polar component. Each tube of dSPE C18 and dSPE C18/PSA was mixed on a vortex mixer for 1 min before centrifugation at 13 000 rpm (5 min). Then, the supernatants (400 μL) were mixed with methanol (100 μL) for LC–MS/MS injection.

#### 2.4.4 Direct Injection Method

Pulverized mushroom (200 mg) was extracted with 1 mL of methanol containing 1% formic acid using a tissue homogenizer at 1200 rpm for 1 min to optimize reproducibility and efficiency with limited sample amounts. The mixture was centrifuged at 13 000 rpm for 5 min (17TR, Hanil Science, Seoul, Korea). The supernatant (80 μL) was mixed with 20 μL of methanol before injection into the LC–MS/MS system.

### 2.5 Instrumental Limit of Quantitation (ILOQ) and Calibration Linearity

The ILOQ and linearity of the method were evaluated using matrix‐matched calibration standards (1–100 ng/mL) prepared in blank shiitake mushroom and human serum matrices. A 5‐μL aliquot of each standard solution, mixed in a 4:1 matrix‐to‐standard ratio, was injected into the LC–MS/MS system. The ILOQ was defined as the lowest concentration at which the signal‐to‐noise (S/N) ratio of the analyte peak exceeded 10. Calibration linearity was assessed in five replicates (*n* = 5), and determination coefficients (*r* ^2^) were calculated.

### 2.6 Accuracy and Precision via Recovery Test

#### 2.6.1 Mushroom Matrix

Blank mushroom samples were fortified with a mixture of α‐amanitin, β‐amanitin, and phalloidin at two levels: the method limit of quantitation (MLOQ) and 10‐fold MLOQ (10 × MLOQ). Each level was prepared in triplicate (*n* = 3) and processed using the procedure described in Section 2.4.4. Recovery rates and precision values were calculated based on LC–MS/MS quantification.

#### 2.6.2 Human Serum Matrix

A human serum sample of 100 μL in a 2‐mL microcentrifuge tube was used for pretreatment, and the following steps were modified with preparation of the mushroom sample (*n* = 3). The 100‐μL human serum sample was added with 100 μL of acidic methanol (1% formic acid) and then homogenized for 1 min at 1200 rpm using a tissue homogenizer (1600 MiniG, SPEX SamplePrep, Metuchen, NJ, USA) for extraction. And then, centrifugation was carried out for 5 min at 13 000 rpm using a centrifuge (17TR, Hanil Science, Seoul, Korea) to separate the layer. The supernatant (80 μL) was then mixed with 20 μL of methanol for matrix matching. Without further cleanup steps, the final matrix‐matched sample was taken into LC–MS/MS for analysis of target analytes.

A 100‐μL aliquot of blank human serum was transferred to a 2‐mL microcentrifuge tube and fortified with the toxin mixture at MLOQ and 10 × MLOQ levels (*n* = 3). For extraction, 100 μL of methanol containing 1% formic acid was added, followed by homogenization for 1 min at 1200 rpm using a tissue homogenizer (1600 MiniG, SPEX SamplePrep, Metuchen, NJ, USA). The mixture was centrifuged at 13 000 rpm for 5 min (17TR, Hanil Science, Seoul, Korea), and 80 μL of supernatant was collected and mixed with 20 μL of methanol for matrix matching. Without additional cleanup, the prepared samples were analyzed directly using LC–MS/MS.

### 2.7 Quantitative Analysis of Field‐Collected Poisonous Mushrooms

Field‐collected specimens of *Amanita virosa* and *Amanita volvata* were analyzed using the validated method. When analyte concentrations exceeded the calibration range, the extracts were diluted accordingly and reanalyzed. To achieve appropriate dilution, 10 μL of *Amanita virosa* extract was mixed with 2.99 mL of matrix‐matched blank shiitake mushroom extract, resulting in a 300‐fold dilution. The diluted mixture was analyzed via LC–MS/MS for quantification of target toxins.

## 3 Results and Discussion

### 3.1 Optimization of UHPLC–MS/MS Conditions

To maximize signal intensity, MRM parameters were optimized by directly infusing each standard solution without an analytical column. Full scan spectra were acquired within the m/z 100–1000 range under simultaneous positive and negative ionization modes. Most analytes showed superior ionization in positive mode, forming [M + H]^+^ ions, whereas [M − H]^−^ ions were also observed in negative mode. Precursor ions were subjected to collision‐induced dissociation, and the most intense product ion was selected as the quantifier. The second most sensitive transition was used as the qualifier. The optimized MRM transitions and corresponding retention times are summarized in Table 1.

**TABLE 1:** Optimized MRM transitions of three mushroom toxins.

No.	Toxin	Monoisotopic mass	Adduct formula	MRM transitions		
				Precursor	Quantifier	Qualifier
1	α‐Amanitin	919.0	[M − H]^−^	917.2	899.1	205.1
2	β‐Amanitin	920.0	[M − H]^−^	918.1	900.1	205.1
3	Phalloidin	788.8	[M + H]^+^	789.1	330.1	157.1

### 3.2 Comparison of Sample Treatment Methods

#### 3.2.1 PRiME HLB Cartridge With and Without LLE

Calibration curves for evaluation of five extraction methods show strong linearity (*r* ^2^ ≥ 0.99), indicating reliable quantitative performance across the tested concentration ranges (Table S1).

Previous studies have demonstrated that acidic conditions improve extraction efficiency for polypeptide toxins such as α‐amanitin, β‐amanitin, and phalloidin [17, 35, 42, 43, 44]. Among various SPE cartridges, C18 and HLB sorbents are frequently applied to purify these hydrophilic toxins in food and biological matrices [21, 22, 23, 24, 35, 45]. PRiME HLB cartridges, based on hydrophilic–lipophilic balance sorbent, offer simplified protocols without the need for conditioning or equilibration.

**FIGURE 3:**
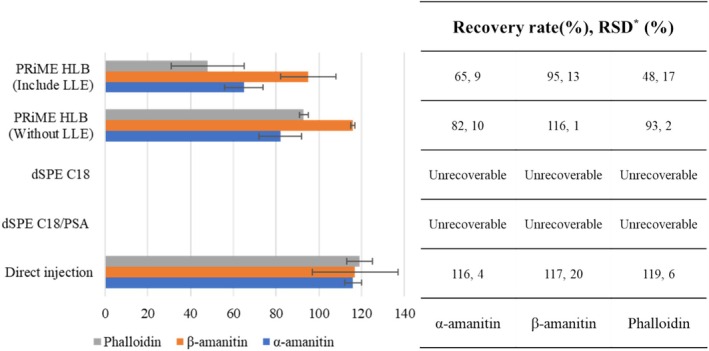
Comparison of recovery rates by five sample preparation methods (concentration: 50 ng/g, *n* = 3). *RSD: relative standard deviation.

In this study, water containing 0.5% hydrochloric acid was used as an extraction solvent, and PRiME HLB cartridges were applied for cleanup. As shown in Figure 3, when LLE was not employed prior to SPE, recovery values for all three toxins ranged from 82% to 116%. In contrast, LLE‐based cleanup resulted in lower and more variable recoveries (48%–95%).

These results indicate that the use of PRiME HLB cartridges without LLE yields higher recovery and lower relative standard deviations (RSDs) for the target analytes. Therefore, a simplified procedure excluding LLE was considered preferable and used as the baseline for further comparison with other methods.

#### 3.2.2 dSPE C18 and dSPE C18/PSA Methods

QuEChERS‐based extraction followed by dSPE cleanup was assessed because of its rapid and straightforward protocol, which is widely utilized for agricultural matrices. Extracts were purified using either dSPE C18 or dSPE C18/PSA and analyzed for recovery of the three toxins.

As shown in Figure 3, the dSPE‐based cleanup conditions employed in this study did not yield acceptable recovery for α‐amanitin, β‐amanitin, and phalloidin. These results indicate that the specific dSPE sorbents and protocols evaluated in this study may not be suitable for extracting highly polar peptide toxins under the tested conditions.

#### 3.2.3 Direct Injection Method

**FIGURE 4:**
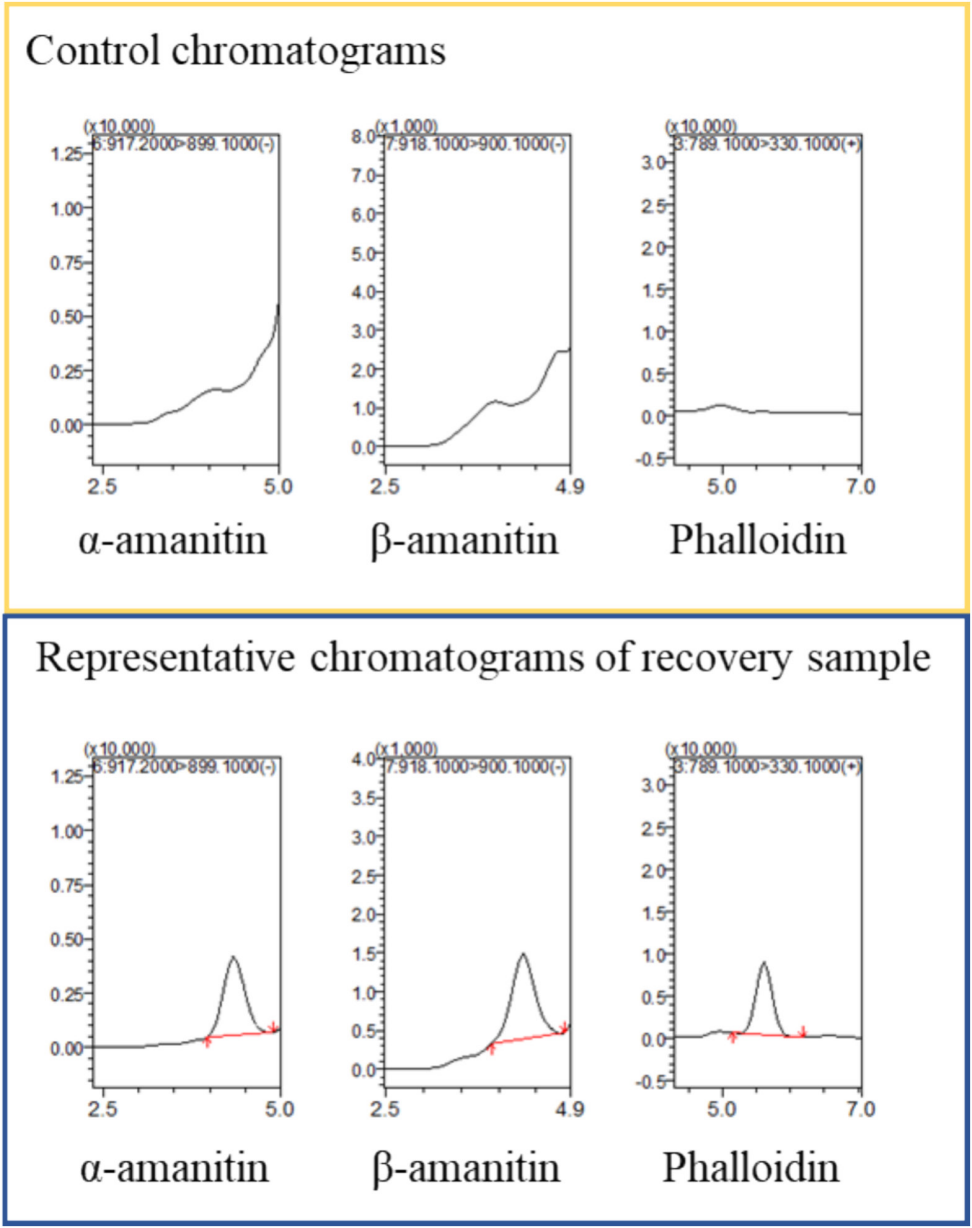
Chromatograms of three mushroom toxins by recovery test.

To further simplify the protocol, direct injection of extracts prepared with methanol containing 1% formic acid was evaluated. Recovery results in Figure 3 demonstrated satisfactory performance, with recovery values ranging from 84% to 120% for all three toxins. Chromatograms obtained during the recovery tests are shown in Figure 4.

As shown in Figure 3, this method not only provided consistent and accurate quantitation but also minimized sample processing time and reduced matrix interference, making it highly suitable for emergency screening or high‐throughput toxicological analysis.

**FIGURE 5:**
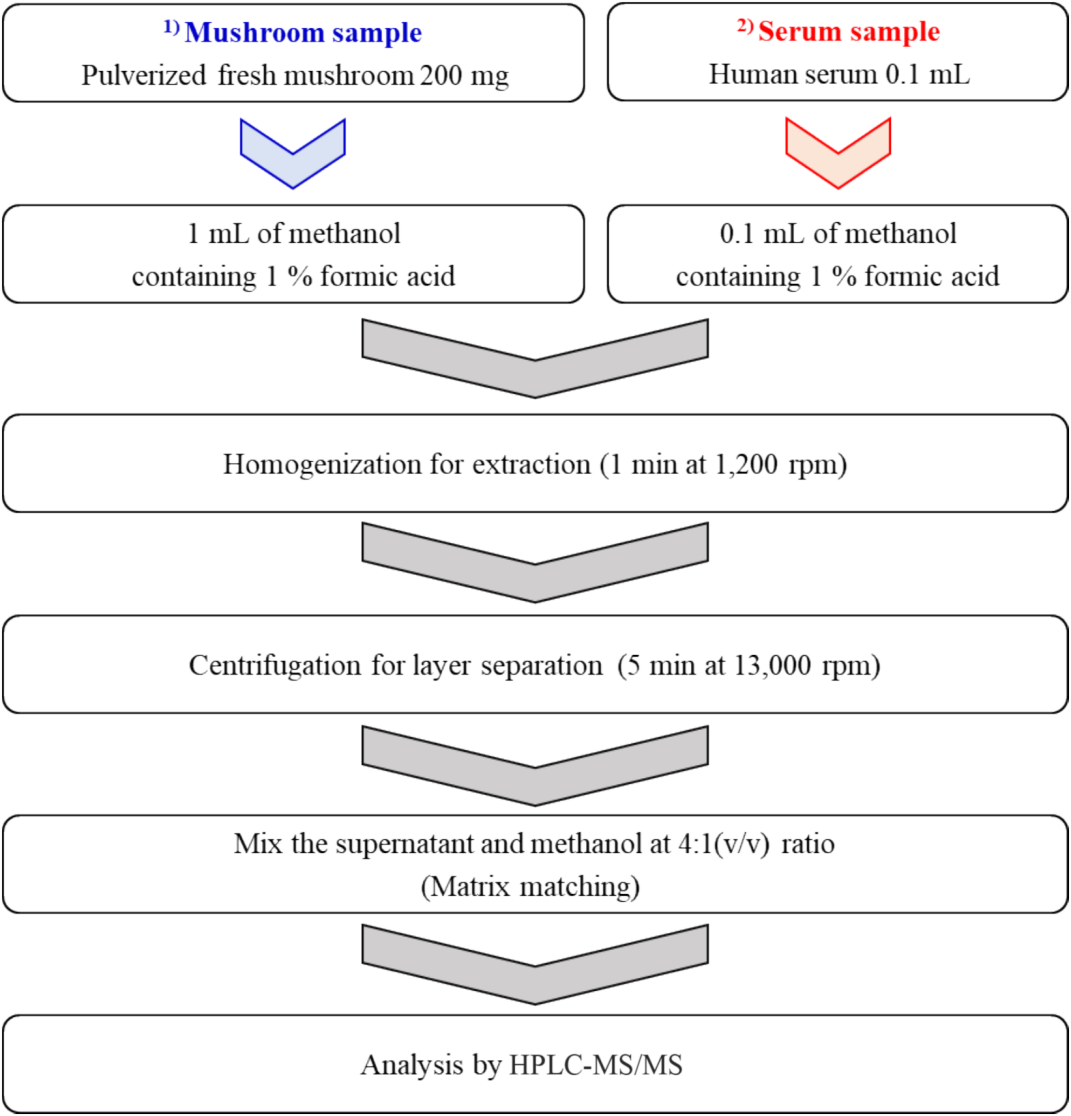
Optimal preparation for mushroom and human serum samples.

Based on these findings, the direct injection protocol was selected as the optimal sample preparation method (Figure 5). This method was subsequently validated using blank mushroom and human serum matrices and applied to the quantitative analysis of field‐collected poisonous mushroom samples.

### 3.3 Validation of Simultaneous Analytical Method

The shiitake mushrooms and human serum were used for validation of the final preparation method. Limit of quantitation (LOQ), linearity of calibration curve, accuracy (recovery, %) and precision (RSD, %) experiments were performed.

ILOQ was defined as the lowest concentration at which the S/N ratio exceeded 10 in the chromatogram. Matrix‐matched calibration curves for α‐amanitin, β‐amanitin, and phalloidin were established over the range from the LOQ to 100 ng/mL, exhibiting strong linearity with determination coefficients (*r* ^2^ ≥ 0.99) in both mushroom and serum matrices (Table 2).

**TABLE 2:** Regression equations for three toxins spiked into shiitake mushrooms and human serum.

Sample	Toxin	Regression equation	Correlation coefficient (*r* ^2^)
Shiitake mushroom	α‐Amanitin	y=6900x−18094	0.9924
	β‐Amanitin	y=2055x−4363	0.9937
	Phalloidin	y=17135x−18483	0.9942
Human serum	α‐Amanitin	y=7099x+2837	0.9911
	β‐Amanitin	y=2237x−262	0.9954
	Phalloidin	y=18021x+13490	0.9962

The ILOQs of α‐amanitin, β‐amanitin, and phalloidin in shiitake mushrooms were 40, 40, and 16 pg, respectively. In human serum, the ILOQ for each toxin was 20 pg. MLOQ values were calculated using the following equation:

$$\text{MLOQ}\;\left(\mathrm{ng}/\mathrm{mL}\right)=\frac{\text{ILOQ}\;\left(\mathrm{pg}\right)}{\text{Injection volume}\;\left(\mathrm{\mu L}\right)}\times \frac{\text{Final sample volume}\;\left(\mathrm{mL}\right)}{\begin{array}{c} \text{Sample volume}\;\left(\mathrm{mL}\right)\\ \text{or sample weight}\;\left(g\right) \end{array}}\times D$$

where *D* is the dilution factor.

The calculated MLOQs in shiitake mushrooms were 50 ng/g for α‐amanitin and β‐amanitin and 20 ng/g for phalloidin. In human serum, the MLOQ was 10 ng/mL for all three toxins.

Recovery and precision were assessed by spiking blank samples at two concentration levels (MLOQ and 10 × MLOQ). The samples were processed using the final optimized method (Figure 5). The results are summarized in Table 3. In mushroom matrices, recovery for the three toxins ranged from 90% to 117% at MLOQ and from 72% to 84% at 10 × MLOQ. Corresponding RSD values were 5%–19% and 2%–5%, respectively. In human serum, recovery ranged from 73% to 79% at MLOQ and from 81% to 95% at 10 × MLOQ, with RSD values of 4%–11% and 3%–7%, respectively. These results indicate that the direct injection method provides acceptable levels of accuracy and precision for quantitative analysis in both mushroom and serum matrices.

**TABLE 3:** Recovery results for three toxins spiked into shiitake mushrooms and human serum (*n* = 3).

Sample	Toxin	Low			High		
		Conc. (ng/g)	Recovery (%)	RSD (%)	Conc. (ng/g)	Recovery (%)	RSD (%)
Shiitake mushroom	α‐Amanitin	50	100	19	500	72	4
	β‐Amanitin	50	90	10	500	82	5
	Phalloidin	20	117	5	200	84	2
Human serum	α‐Amanitin	10	79	5	100	91	7
	β‐Amanitin	10	74	11	100	88	3
	Phalloidin	10	73	4	100	81	5

Abbreviation: RSD: relative standard deviation.

### 3.4 Quantitative Analysis of Toxins in Field‐Collected Mushrooms

**FIGURE 6:**
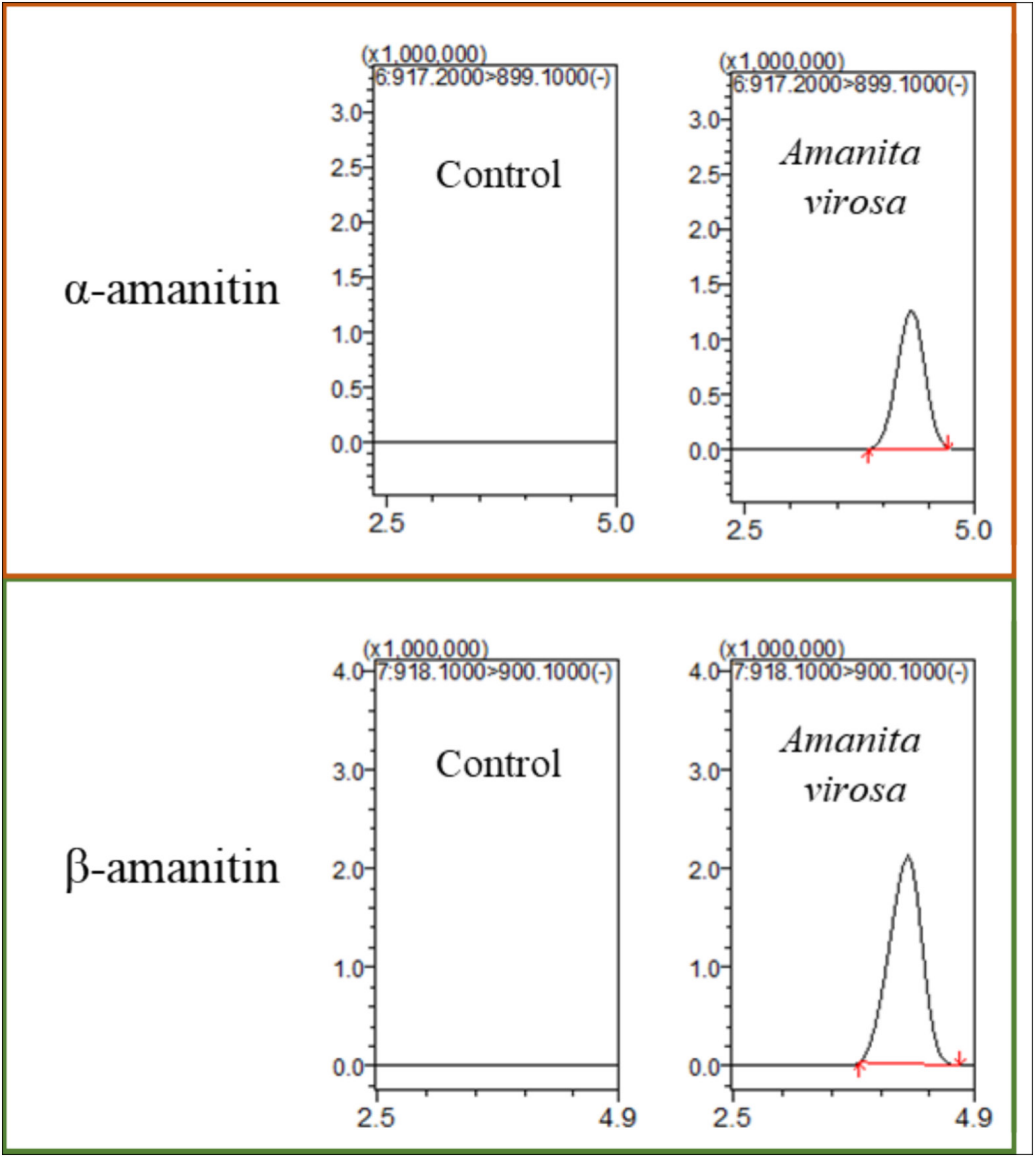
Chromatograms for detected toxins in actual *Amanita virosa*.

The validated method was applied to the quantitation of α‐amanitin, β‐amanitin, and phalloidin in field‐collected specimens of *Amanita virosa* and *Amanita volvata*. Previously reported concentration ranges for these toxins in *Amanita* species vary from 20 to 9311 μg/g (Table 4), suggesting that the developed method possesses sufficient sensitivity for real sample analysis [32]. The results of quantitative analysis were presented in Table 5, and representative chromatograms are shown in Figure 6. In *Amanita virosa*, α‐amanitin and β‐amanitin were detected at 39 and 145 mg/kg, respectively, whereas phalloidin was not detected. In contrast, all target toxins were below the detection limit in *Amanita volvata*. These findings are consistent with previous studies reporting concentration ranges of 60–483 mg/kg for α‐amanitin, 63–363 mg/kg for β‐amanitin, and 92–241 mg/kg for phalloidin in *Amanita* species (Table 5).

**TABLE 4:** Concentrations of three toxins in *Amanita*.

Species	Region	α‐Amanitin	β‐Amanitin	Phalloidin	Ref.	
*A. phalloides*	Turkey	10–39	4–33	NM	5	
*A. phalloides*	Portugal	666–784	NM	NM	37	
*A. phalloides*	Italy	1330	NM	NM	38	
*A. phalloides*	United States	880	NM	NM	38	
*A. phalloides* var. *alba*	Turkey	2140	1710	2100	46	
*A. phalloides* ^a^	United Kingdom	60	63	92	42	
*A. phalloides* ^a^	France	113	123	106	32	
*A. fuliginea*	China	2500–7000	810–1890	780–1350	47	
*A. virosa*	Italy	1390	NM	NM	38	
*A. virosa*	Japan	983	228	328	24	
*Amanita* (24)	Colombia	50–6000	NM	NM	48	
*A. virosa* ^a^	Japan	52–483	27–363	95–241	17	
*A. virosa*	China	2834	NM	NM	49	
*Amanita* (28)	China	20–9311	33–1758	379–1464	50	
Range (mg/kg)	—	10–9311	4–1890	92–1464	—	

*Note:* Numbers in parentheses in Column 1 indicate the number of *Amanita* species.

Abbreviation: NM: not mentioned.

^a^ Fresh sample.

**TABLE 5:** Concentration of three toxins in actual *Amanita virosa* and *Amanita volvata* (*n* = 3).

Poisonous mushroom	Concentration of toxin (mg/kg)		
	α‐Amanitin	β‐Amanitin	Phalloidin
*Amanita virosa*	39	145	ND
*Amanita volvata*	ND	ND	ND

Abbreviation: ND: not determined.

## Conclusions

A rapid and sensitive analytical method was developed and validated for the simultaneous determination of α‐amanitin, β‐amanitin, and phalloidin in mushroom and human serum matrices using UHPLC–MS/MS. Among the evaluated sample preparation strategies, the direct injection method using methanolic extraction with 1% formic acid demonstrated optimal performance, offering acceptable recovery, precision, and minimal matrix interference. The method achieved low limits of quantitation (LOQ) ranging from 10 to 50 ng/mL depending on the matrix and analyte, with excellent linearity (*r* ^2^ ≥ 0.99) in both mushroom and serum matrices. Validation results confirmed the applicability of the method for trace‐level quantitation of peptide toxins in complex biological and food matrices. The established protocol was successfully applied to the analysis of field‐collected *Amanita virosa* and *Amanita volvata*, detecting α‐amanitin and β‐amanitin in *Amanita virosa* at concentrations consistent with previous reports. This method provides a practical and efficient analytical platform suitable for forensic, clinical, and food safety applications, particularly under time‐sensitive conditions such as acute poisoning cases.

## Author Contributions

H.J.O. and E.Y.P.: data curation, formal analysis, investigation, validation, and writing—original draft. Y.H.S.: methodology. J.H.K.: supervision. M.H.S. and J.H.L.: project administration, conceptualization, supervision, writing—original draft, and writing—review and editing. All the authors have read and approved the final manuscript.

## Conflicts of Interest

The authors declare no conflicts of interest.

## Supporting information


**Table S1** Regression equations and correlation coefficients for evaluation of five sample preparation techniques.

## Data Availability

All data generated or analyzed during this study are included in this published article.
