# Supplementary material for: Development of Simultaneous Analytical Method of Three Polypeptide Toxins α‐Amanitin, β‐Amanitin and Phalloidin in Poisonous Mushrooms and Human Serum Using UHPLC–MS/MS
Source: J Mass Spectrom. 2025 May 21;60(6):e5145. doi: 10.1002/jms.5145 (PMC12093918; doi:10.1002/jms.5145)
Supplement: Supplementary file 1 — Table S1 Regression equations and correlation coefficients for evaluation of five sample preparation techniques. [file JMS-60-e5145-s001.docx]

**Supplemental data**

**Development of simultaneous analytical method of three polypeptide toxins
α-amanitin, β-amanitin and phalloidin in poisonous mushrooms and human serum using UHPLC-MS/MS**

Hang-Ji Ok^1,2†^, Eun-Young Park^3†^, Yongho Shin^4^, Jeong-Han Kim^1^, Min-Ho Song^5*^, and Ji-Ho Lee^5*^

*^1^Department of Agricultural Biotechnology of Agriculture and Life Sciences, Seoul National University, Seoul, Republic of Korea*

*^2^Department of Technical Research Center, Shimadzu Scientific Korea, Seoul, Republic of Korea*

*^3^Department of Food Science and Technology, University of California Davis, Davis, CA, United States*

*^4^Department of Applied Bioscience, Dong-A University, Busan, Republic of Korea*

*^5^* *School of Natural Resources and Environment Science College of Agriculture and Life Sciences, Kangwon National University, Gangwon State, Republic of Korea**Corresponding author:

^*^Min-Ho Song

E-mail: [minobel@naver.com](mailto:minobel@naver.com)

Phone No.: +82-10-4731-6187

^*^Ji-Ho Lee

E-mail: [micai@kangwon.ac.kr](mailto:micai@kangwon.ac.kr)

Phone No.: +82-10-2012-5548

Table S1. Regression equations and correlation coefficients for evaluation of five sample preparation techniques

| **Sample** | **Toxin** | **Regression equation** | **Correlation coefficient (r^2^)** |
| --- | --- | --- | --- |
| PRiME HLB  (Include LLE) | α-amanitin | y = 8413x + 34359 | 0.9965 |
|  | β-amanitin | y = 4650x + 20325 | 0.9983 |
|  | phalloidin | y = 1580x + 31640 | 0.9944 |
| PRiME HLB  (Without LLE) | α-amanitin | y = 7910x + 14575 | 0.9421 |
|  | β-amanitin | y = 2251x + 72646 | 0.9691 |
|  | phalloidin | y = 742x + 19730 | 0.9994 |
| dSPE C18 | α-amanitin | y = 11800x - 14355 | 0.9990 |
|  | β-amanitin | y = 3146x - 7848 | 0.9978 |
|  | phalloidin | y = 1015x - 3542 | 0.9995 |
| dSPE C18/PSA | α-amanitin | y = 11212x - 18382 | 0.999 |
|  | β-amanitin | y = 3515x - 8306 | 0.9983 |
|  | phalloidin | y = 1164x - 4751 | 0.9969 |
| Direct injection method | α-amanitin | y=17135x - 18483 | 0.9921 |
|  | β-amanitin | y=6900x - 18094 | 0.9877 |
|  | phalloidin | y=2055x - 4363 | 0.9981 |
